# Supplementary material for: Acceptance and compliance with micronutrient powder (MNP) among children aged 6–23 months in northern Nigeria
Source: PLOS Glob Public Health. 2022 Oct 17;2(10):e0000961. doi: 10.1371/journal.pgph.0000961 (PMC10022258; doi:10.1371/journal.pgph.0000961)
Supplement: S1 Dataset — (ZIP) [file pgph.0000961.s008.zip › S8_Dataset/Spot Checks.zip!/Spot Checks/12072015_A_GU_PM_2_SP.pdf]

SPOT CHECK RECORDING FORM

State: ____ADAMAWA____________ LGA: _____GUYUK____________ Community: ____LAKUMNA_________________

Sex of index child: ___FEMALE___________ Age of index child (in months): ____6______________

Spot Check Information

1. **Date of MNP distribution to household** (circle option below based on LGA name above)**:**
   - **Kebbi:** BirninKebbi&Jega LGA: **June 16**
   - **Kebbi:** Danko-wasagu: **June 17**
   - **Adamawa:**Song, Ganye: **June 18**
   - **Adamawa:**Guyuk **June 19**
2. **Date of Spot Check Observation: _12-07-15__________________**
3. **Number of sachets remaining**(Ask the caregiver to show you and count the specific number of sachets remaining)**:**

34

4. **Number of sachets that should remainusing one MNP sachet per day** (Analyst only)**:**

38

5. **Number of sachets remaining / number of sachets should be remaining**(Analyst only)**:**

   ____34_____ / ___38______ = ____89.5_____ %

Probes

Question 1. You have **XX** MNP sachets remaining. Explain how easy or difficult it was to use the MNP product on a daily basis for your child 6 – 23 months? (Circle one answer)

Very Difficult **Difficult** **Easy** **Very Easy**

Question 2. Describe any specific challenges that you faced that made the MNP difficult to use on a daily basis?NO CHALLENGES AT ALL.

Question 3. Describe specific aspects of the MNP product that you or your child enjoyed that made it easier to use on a daily basis? WELL,ME PERSONALLY,I VIEW THE POWDER AS A VACCINATION AGAINST DISEASES AND ILL HEALTH TO A CHILD…..THAT IS THE MOTIVATION THAT ENCOURAGES ME TO ADMINISTER THE MNP ON A DAILY BASIS WITHOUT DEFAULTING.

Question 4. What questions do you have about the MNP based on your experience using it? **NO I DON'T HAVE ANY QUESTION IN SHORT,ME AM VERY HAPPY WITH THE MNP,I WOULD BE VERY GLAD TO USE IT ANYTIME,THAT IS WHY I NEVER MISSED A DAY SINCE I WAS GIVEN THE POWDER.**

Question 5. What specific recommendations do you have that we can do as a program to make the MNP easier to use on a daily basis? I CANT THINK OF ANY FOR NOW.
